# PET/MRI in prostate cancer: a systematic review and meta-analysis

**DOI:** 10.1007/s00259-020-05025-0

**Published:** 2020-09-08

**Authors:** Laura Evangelista, Fabio Zattoni, Gianluca Cassarino, Paolo Artioli, Diego Cecchin, Fabrizio dal Moro, Pietro Zucchetta

**Affiliations:** 1 Nuclear Medicine Unit, Department of Medicine, Padova University Hospital, Via Giustiniani 2, Padova, Italy; 2 Urology Unit, Department of Medicine, Udine University Hospital, Udine, Italy; 3 Urology Unit, Department of Surgery, Oncology and Gastroenterology, University of Padova, Padova, Italy

**Keywords:** Prostate cancer, Positron emission tomography, Magnetic resonance imaging, PSMA, Choline

## Abstract

**Aim:**

In recent years, the clinical availability of scanners for integrated positron emission tomography (PET) and magnetic resonance imaging (MRI) has enabled the practical potential of multimodal, combined metabolic-receptor, anatomical, and functional imaging to be explored. The present systematic review and meta-analysis summarize the diagnostic information provided by PET/MRI in patients with prostate cancer (PCa).

**Materials and methods:**

A literature search was conducted in three different databases. The terms used were “choline” or “prostate-specific membrane antigen - PSMA” AND “prostate cancer” or “prostate” AND “PET/MRI” or “PET MRI” or “PET-MRI” or “positron emission tomography/magnetic resonance imaging.” All relevant records identified were combined, and the full texts were retrieved. Reports were excluded if (1) they did not consider hybrid PET/MRI; or (2) the sample size was < 10 patients; or (3) the raw data were not enough to enable the completion of a 2 × 2 contingency table.

**Results:**

Fifty articles were eligible for systematic review, and 23 for meta-analysis. The pooled data concerned 2104 patients. Initial disease staging was the main indication for PET/MRI in 24 studies. Radiolabeled PSMA was the tracer most frequently used. In primary tumors, the pooled sensitivity for the patient-based analysis was 94.9%. At restaging, the pooled detection rate was 80.9% and was higher for radiolabeled PSMA than for choline (81.8% and 77.3%, respectively).

**Conclusions:**

PET/MRI proved highly sensitive in detecting primary PCa, with a high detection rate for recurrent disease, particularly when radiolabeled PSMA was used.

**Electronic supplementary material:**

The online version of this article (10.1007/s00259-020-05025-0) contains supplementary material, which is available to authorized users.

## Introduction

The availability of tracers other than 18f-fluorodeoxyglucose (FDG) suggests new opportunities for the diagnosis and management of prostate cancer (PCa). The use of different radiopharmaceuticals, such as radiolabeled choline, or radiolabeled ligands of prostate-specific membrane antigen (PSMA), has a significant impact in various clinical settings, from initial staging to the detection of a biochemical recurrence, enabling personalized treatment planning, and metastasis-directed therapy (MDT) [1, 2]. Such an approach relies on the diagnostic performance of the imaging modalities used to detect the real extent and location of metastases. Many studies on PCa patients have been conducted using PET/CT [3–5], but most clinical protocols consider magnetic resonance imaging (MRI) the principal imaging modality for staging and restaging of patients with PCa.

In recent years, the clinical availability of integrated PET/MRI scanners has made it possible to explore the practical potential of multimodal, combined metabolic-receptor, anatomical, and functional imaging. The present systematic review and meta-analysis summarize the diagnostic information obtained with PET/MRI in PCa patients.

## Materials and methods

### Search strategy and study selection

A literature search from 2013 up to 23rd March 2020 was conducted in the PubMed, Scopus, and Web of Science databases. The terms used were as follows: “choline” or “PSMA” AND “prostate cancer” or “prostate” AND “PET/MRI” or “PET MRI” or “PET-MRI” or “positron emission tomography/magnetic resonance imaging.” The search was carried out with and without the addition of filters, such as English language only, type of article (original article, research article), and subjects (humans only). Three reviewers (L.E., F.Z., and P.A.) conducted the literature search, and two other reviewers (G.C. and D.C.) independently selected the studies to consider, excluding duplicate papers. Any discrepancy was solved by a consensus. After combining all the records identified, the full texts were retrieved and further assessed by four of the reviewers (F.Z, P.A., G.C., and L.E.).

One reviewer (L.E.) ran a new search across the databases, checking the references of the studies already selected, to ensure their eligibility. Reviews, clinical reports, abstracts of meetings, and editorials were excluded. The qualitative analysis excluded reports that did not consider hybrid PET/MRI scanners or that enrolled a very low number of patients (< 5). Studies were eligible for inclusion in the meta-analysis if all the following requirements were met: (i) a sample size of more than ten patients; and (ii) the article included enough raw data to enable the completion of a 2 × 2 contingency table (or the authors made said data available on request).

### Data extraction

General details were retrieved for each study considered, such as generic data (authors, journal name, year of publication, country, and study design), patients’ characteristics (number of patients and their mean or median age), disease phase (i.e., staging or restaging), type of treatment, mean or median PSA level at the time of PET, and radiotracer used for PET/MRI. A quality assessment on the studies was performed using the Quality Assessment of Diagnostic Accuracy Studies 2 (QUADAS-2) [6]. Data extraction and quality assessment were done independently by three reviewers (L.E., F.Z., G.C.), and differences were solved by discussion.

### Statistical methods

The pooled detection rate of PET/MRI, with its sensitivities, specificities, and 95% confidence intervals (CIs), with both radiolabeled choline and radiolabeled PSMA, was calculated using random effects analysis. Heterogeneity was tested using the *χ*^2^ and the *I*^2^ tests. The *χ*^2^ test provided an estimate of the between-study variance, and the *I*^2^ test measured the proportion of inconsistency in individual studies that cannot be explained by chance. The values for heterogeneity (*I*^2^) of 25%, 50%, and 75% were considered low, moderate, and high, respectively [7]. Publication bias was assessed using Deeks’ funnel plot asymmetry test, and a *P* value above 0.05 suggested the absence of any publication bias. All statistical analyses were performed using the Meta-DiSc® version 1.4 (developed by the Clinical Biostatistics Unit at Ramón y Cajal Hospital, Madrid) and Comprehensive Meta-Analysis (CMA) software version 3.3.070 (Biostat, Englewood, NJ, USA).

## Results

### Qualitative results

**Fig. 1:**
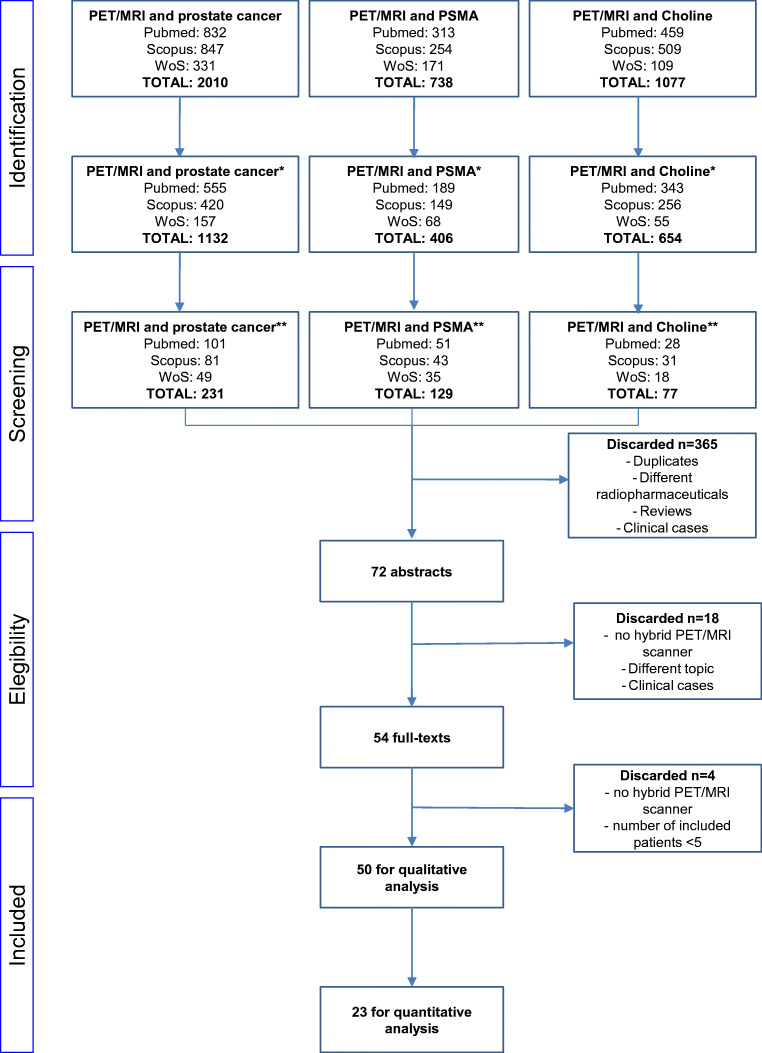
The PRISMA method for study selection (*filters, journal article/humans/last 5 years/English language; **exclusion of reviews, no inclusion of PET/MRI in the title and exclusion of clinical case)

In total, 50 studies were eligible for qualitative analysis (Fig. 1, Table 1), 20 of them were prospective, and 30 were retrospective. Overall, the analysis concerned 2059 patients who underwent hybrid PET/MRI. Disease staging was the most common reason for the test (*n* = 24 studies; totally, 940 patients), followed by restaging (*n* = 16; totally 844 patients), and both staging and restaging (*n* = 10; totally 275 patients). Radiolabeled PSMA was used in the majority of cases (*n* = 34 studies). In 25 studies, the main endpoint was the ability of PET/MRI to detect PCa, be it primary or recurrent disease. Comparisons were drawn between PET/CT and PET/MRI performed in the same populations in 7 reports.

**Table 1:** Characteristics of the selected studies

Authors	Ref	Year of pub	Country	Retrospective vs. prospective study design	Age (median or mean ± SD) in years	*N* of pts	Mean-median PSA (SD-IQR)	Setting of disease	Treatments before PET	RA	Number of pts undergoing PET/MRI	Study content	Outcome
Afshar-Oromieh et al.	[8]	2013	Germany	Prospective	69.6(± 7.3)	20	2.62 ng/mL (0.5–73.60)	Restaging	RP, RP+RT, RP+ADT, RT+ADT	68Ga-PSMA-11	20	PET/CT vs. PET/MRI	PCa was detected more easily and more accurately with Ga-PSMA PET/MRI than with PET/CT and with lower radiation exposure.
Wetter et al.	[9]	2013	Germany	Prospective	74 (59–85)	55	NA	Restaging	NA	18F-choline	55	Imaging interpretation	Inverse correlation between increased choline metabolism and ADC values of bone metastases.
Souvatzoglou et al.	[10]	2013	Germany	Prospective	69.2 ± 5.7	32	5.5 ± 7.3 ng/mL	Staging and restaging	RP, RT, HIFU, ADT plus CT	11C-Choline	32	PET/CT vs. PET/MRI	The better anatomical allocation of intraprostatic and bone lesions by PET/MRI than by PET/CT raises the expectation that simultaneous PET/MRI may improve diagnostic performance in the evaluation of PCa.
Wetter et al.	[11]	2013	Germany	Prospective	69.5 (56–85)	36	NA	Staging	RP, RT, and ADT	18F-Choline	36	Detection of disease	Integrated PET/MRI performed with a dedicated integrated PET/MRI scanner is expected to provide reasonable accuracy and diagnostic performance in the detection/localization of PCa. In low GS patients, it would replace PET/CT and mpMRI for the initial staging of disease.
Wetter et al.	[12]	2014	Germany	Prospective	64 (49–80)	35	25.7 ± 23.1 ng/mL	Staging	None	18F-choline	21	Imaging interpretation	Both SUVs and ADC values differ significantly between tumor lesions and healthy tissue. However, there is no significant correlation between these two parameters. This might be explained by the fact that SUVs and ADC values characterize different parts of tumor biology.
de Perrot et al.	[13]	2014	Switzerland	Prospective	NA	26	NA	Staging	RP	18F-Choline	26	Detection of disease	PET/MRI allowed precise localization of foci in the prostate.
Kim et al.	[14]	2015	Korea	Prospective	69.4 ± 6.7	30	14.9 ± 15.1 ng/mL	Staging	NA	18F-Choline	30	Detection of disease	Simultaneous PET/MRI is better for the detection of cancer than each individual modality. New MRI-assisted metabolic volumetric parameters provide better characterization of primary prostate cancers than conventional PET and MRI parameters.
Gatidis et al.	[15]	2015	Germany	Prospective	67 ± 10	16	NA	Staging and restaging	None, TURP, RT	11C-Choline	16	Technical aspect	The combined sFCM/SVM algorithm proposed in this study revealed reliable classification results consistent with the histopathological reference standard and comparable with those of manual tumor delineation. sFCM/SVM generally performed better than unsupervised sFCM alone.
Freitag et al.	[16]	2015	Germany	Retrospective	66	26	15.95 ng/mL	Staging	NA	68Ga-PSMA-11	26	PET/CT vs. PET/MRI	Lymph node and osseous metastases of PCa are accurately and reliably depicted by PET/MRI with very high concordance 98.5% compared with PET/CT including PET-positive LNs of normal size. For both lymph nodes and bone metastases, T2-w fat-saturated and DWIb800 sequences provided the best visibility scores for anatomical correlation.
Wetter et al.	[17]	2017	Germany	Retrospective	68.1 (± 7.9)	22	27.6 ± 4.1 ng/mL	Staging	None	18F-Choline	20	Imaging interpretation	Simultaneous acquisition of PET and MR spectroscopy with integrated PET/MRI is feasible. Choline compounds and choline metabolism show a positive significant correlation.
Eiber et al.	[18]	2016	Germany	Retrospective	NA	66	12.0 ng/mL (6.9–18.8)	Staging	NA	68Ga-PSMA-11	53	Detection of disease	Simultaneous 68Ga-PSMA-11 PET/MRI improves diagnostic accuracy for PCa localization both compared with mpMRI and with PET imaging alone.
Lutje et al.	[19]	2016	Germany	Retrospective	69	20	19.0 ng/mL	Staging and restaging	NA	68Ga-PSMA-11	20	Acquisition protocol	PET image quality obtained with PET/MRI using 68Ga-PSMA-11 ligands reaches its maximum around an acquisition time of 4 min.
Domachevsky et al.	[20]	2017	Israel	Retrospective	67.1 ± 12.1	21	NA	Staging and restaging	NA	68Ga-PSMA-11	21	PET/CT vs. PET/MRI	Early PET/MRI demonstrates very good lesion detectability agreement and correlation with PET metrics compared with same day PET/CT.
Eiber et al.	[21]	2017	Germany	Retrospective	70 (51–85)	75	2.6 ng/mL (0.2–88)	Restaging	RP, RT and ADT	11C-choline	75	PET/CT vs. PET/MRI	PET/MRI has a higher diagnostic value for detecting local recurrence compared with PET/CT with the advantage of substantial dose reduction. Use of 11C-choline PET/MRI especially for patients with low (≤ 2 ng/mL) PSA values, whereas PET/CT is preferable in the subgroup with higher PSA values.
Heußer et al.	[22]	2017	Australia	Retrospective	NA	31	NA	Staging and restaging	NA	68Ga-PSMA-11	31	Technical aspect	Halo artifacts can be reduced by reducing the maximum scatter faction rate.
Lake et al.	[23]	2017	USA	Retrospective	68.3 (6.9)	55	7.9 ng/mL (12.9)	Restaging	RP, RT, RP+RT	68Ga-PSMA-11	55	Acquisition protocol	The 8-min PET acquisition was superior to the 3-min acquisition for detection of small lymph nodes.
Noto et al.	[24]	2017	Germany	Retrospective	65.3 ± 9.3	12	NA	Staging and restaging	NA	68Ga-PSMA-11	12	Acquisition protocol	Short acquisition durations of less than 3 min per bed position result in unacceptable image artifacts and decreased diagnostic performance in current whole-body 68Ga-PSMA PET/MRI and should be avoided.
Lütje et al.	[25]	2017	Germany	Prospective	70.5 (56–83)	44	3.9 ng/mL (0–10)	Restaging	RP	68Ga-PSMA-11	25	PET/CT vs. PET/MRI	68Ga-PSMA 11 PET/MRI is superior to PET/CT.
Hope et al.	[26]	2017	USA	Prospective	69 ± 6.9	150	5.9 ± 10.3 ng/mL	Restaging	RP, RT ± ADT, RP + RT	68Ga-PSMA-11	63	Detection of disease	68Ga-PSMA-11 PET has a high detection rate that resulted in a major change in management in 53% of patients with BCR in our study.
Bates et al.	[27]	2017	Australia	Prospective	65 (51–80)	30	NA	Staging	TRUS and RP	68Ga-PSMA-11	30	Imaging interpretation	Association between abnormal expression of PSMA within the prostatic transition zone and altered texture on T2-weighted MRI.
Schiller et al.	[28]	2017	Germany	prospective	64 (49–76)	31	15.7 ng/mL (4.3–56)	Restaging	NA	68Ga-PSMA-11	10	Detection of disease	Compared with conventional CT or MRI staging, 68Ga-PSMA PET imaging detects more PC lesions and, thus, significantly influences radiation planning in recurrent PCa patients enabling individually tailored treatment.
Lee et al.	[29]	2017	Korea	Prospective	68.3 (64.6–72.8)	35	20.14 ng/mL (3.33–66.95)	Staging	NA	18F-Choline and 18F-FDG	31	Detection of disease	PET/MRI has a better sensitivity than mpMRI.
Freitag et al.	[30]	2017	Germany	retrospective	NA	119	1.70 ng/mL (1.25–2.20)	Restaging	PR	68Ga-PSMA-11	93	PET/CT vs. PET/MRI	Additional value of hybrid 68Ga-PSMA-11-PET/MRI by gaining complementary diagnostic information compared with the 68Ga-PSMA-11 PET/CT.
Bauman et al.	[31]	2018	Canada	Prospective	63 (58.5–66.5)	6	8.45 ng/mL (4.5–16.0)	Staging	None	18F-DCFPyL	6	Detection of disease	PET/MRI was able to identify locations of prostate cancer in the prostate glands of men undergoing imaging before surgery.
Kranzbühler et al.	[32]	2018	Switzerland	Retrospective	69 (11)	56	0.99 ng/mL (3.1)	Restaging	RP(plus RT, plus ADT)	68Ga-PSMA-11	56	Detection of disease	PET/MRI has a high detection rate for recurrent prostate cancer even at very low PSA levels < 0.5 ng/mL. Furthermore, even at those low levels, extrapelvic disease can be localized in 25% of the cases, and local recurrence alone is seen only in 10%.
Freitag et al.	[33]	2018	Germany	Retrospective	71.5 (64.5–73.0)	8	7.3 ng/mL (2.5–8.6)	Staging andrestaging	RP, RP+RT	18F–PSMA-1007	8	Detection of disease	PET/MRI combines efficient whole-body assessment with high-resolution co-registered PET/MRI of the prostatic fossa for comprehensive oncological staging of patients with PCa.
Grubmüller et al.	[34]	2018	Austria	Retrospective	74 (68–76)	117	1.04 ng/ml (IQR 0.58–1.87)	Restaging	RP, RP+RT	68Ga-PSMA 11	71	Detection of disease	High performance of PSMA PET imaging for the detection of disease recurrence sites. It adds significant information to standard CT/MRI, changing treatment strategies in a significant number of patients.
Al-Bayati et al.	[35]	2018	Germany	Retrospective	68.2 ± 8.5	22	14.5 ± 13.0 ng/mL	Staging	None	68Ga-PSMA-11	22	Detection of disease	PET/MRI demonstrates higher diagnostic accuracy than mpMRI and is particularly valuable in tumors with equivocal results from PI-RADS classification.
Pizzuto et al.	[36]	2018	Switzerland	Retrospective	63 (7)	31	12.6 (16) ng/mL	Staging	None	68Ga-PSMA-11	31	Imaging interpretation	Higher 68Ga-PSMA-11 accumulation in the central zone than in the transition and peripheral zones is normal, and leads to a pattern resembling Mickey Mouse ears on 68Ga-PSMA-11 PET. This pattern could be helpful in avoiding false-positive interpretations of PET scans.
Taneja et al.	[37]	2018	India	Retrospective	64.9 ± 1.5	35	NA	Staging	None	68Ga-PSMA-11	35	Acquisition protocol	Dual-phase PSMA uptake improves accuracy of classifying malignant vs. benign prostate lesions and complements multiparametric MRI in the diagnosis of PCa.
Park et al.	[38]	2018	USA	Prospective	66.4 (55–74)	33	9.6 ng/mL (5.8)	Staging	None	68Ga-PSMA-11	33	Detection of disease	PET can be used to identify prostate cancer, while MRI provides detailed anatomic guidance. Hence, 68Ga-PSMA-11 PET/MRI provides valuable diagnostic information and may inform the need for and extent of pelvic node dissection.
Riola-Parada et al.	[39]	2018	Spain	Retrospective	71.25 (56–71)	27	2.94 ng/mL (0.18–10)	Restaging	RP, RT, BRT, RP+RT, BT+RT, cryotherapy and HIFU	18F-choline	27	Detection of disease	18F-choline PET/MRI detection rate was considerable despite the relatively low PSA values in our sample. The influence of Gleason score and PSA level on 18F-choline PET/MRI detection rate was not statistically significant.
Thalgott et al.	[40]	2018	Germany	Retrospective	68 (IQR: 63–73)	102	14.0 ng/mL (IQR: 6–35)	Staging	None	68Ga-PSMA-11	73	Detection of disease	PET/MRI performs at least equally for tumor and lymph node stage prediction compared with nomograms in high-risk PCa patients.
Muehlematter et al.	[41]	2018	Switzerland	Prospective	72.5 (60–89)	20	NA	Staging and restaging	NA	18F-choline	20	Technical aspect	Addition of TOF information has a positive impact on lesion detection rate for lymph node and bone metastasis in PCa.
Ferda et al.	[42]	2018	Czech Republic	Retrospective	63.2 (47–78)	100	NA	Restaging	NA	18F-choline	100	Detection of disease	PET/MRI with 18F-choline is a valuable tool in evaluation of restaging in patients with PCa, with high detection rate even in those with a low serum PSA level.
Tseng et al.	[43]	2018	Taiwan	Retrospective	70 (52–84)	31	30.56 ng/mL (47.5–591.9)	Staging	None	11C-Choline	31	Imaging interpretation	Semiquantitative PET and MRI data are connected with the prognosis.
Jena et al.	[44]	2018	India	Retrospective	64 ± 1	82	NA	Staging	None	68Ga-PSMA-11	82	Detection of disease	High diagnostic accuracy in primary tumors by using PET/MRI.
Hicks et al.	[45]	2018	USA	Retrospective	68 (62–71)	32	13.4 ng/mL (8.4–19.7)	Staging	None	68Ga-PSMA-11	32	Detection of disease	Accuracy of PET/MRI for the primary tumor is higher than mpMRI alone.
Grubmuller et al.	[46]	2018	Austria	Prospective	64 (59–71)	122	7.63 ng/mL (5.5–13.4)	Staging	None	68Ga-PSMA-11	122	Detection of disease	PET/MRI is accurate in the initial staging and it can change the management.
Ferraro et al.	[47]	2019	Switzerland	Retrospective	65 (51–79)	60	13 ± 13.6 ng/mL	Staging	None	68Ga-PSMA-11	46	Detection of disease	Data from PET are able to select patients who benefit from ePLND.
Ettala et al.	[48]	2019	Finland	Prospective	71 (64–78)	9	52 ng/mL (7–280)	Staging	ADT	68Ga-PSMA-11	9	Acquisition protocol	68Ga-PSMA is associated with an increase uptake due to ADT administration. The optimal time to acquisition is after 3–4 weeks post-ADT.
Uslu-Besli et al.	[49]	2019	Turkey	Retrospective	67.9 ± 7.0	26	65.2 ± 199.6 ng/mL	Staging	None	68Ga-PSMA-11	26	Imaging interpretation	Correlation between SUVmax and ADC in the primary PCa.
Bialek et al.	[50]	2019	Poland	Retrospective	64.4 ± 7.07	89	NA	Staging and restaging	NA	68Ga-PSMA-11	89	Imaging interpretation	Cervical sympathetic ganglia should not be falsely interpreted as laterocervical lymph nodes.
Abufaraj et al.	[8]	2019	Austria	Prospective	61 (59–66)	65	9 ng/mL (7–12)	Restaging	RP, ADT and RT	68Ga-PSMA-11	65	Detection of disease	PET/MRI has a good performance for the identification of metastatic lymph nodes.
Achard et al.	[51]	2019	Switzerland	Retrospective	67 (47–83)	53	1.5 ng/mL (0.1–31.8)	Restaging	RP	18F-Choline	53	Detection of disease	18F-Choline PET/MRI has an important impact on the detection rate and management of patients with recurrent PCa.
Burger et al.	[52]	2019	Switzerland	Prospective	68 ± 4.3	10	3.1 ± 2.2 ng/mL	Restaging	HIFU	68Ga-PSMA-11	10	Detection of disease	PSMA PET/MRI can detect the presence of recurrence after HIFU in patients with a negative mpMRI.
Metser et al.	[53]	2019	Toronto, Canada	Prospective	NA	58	NA	Staging	None	18F-Choline	58	Acquisition protocol	Technical information about the type of PET/MRI protocol.
Muehlematter et al.	[54]	2019	Switzerland	Retrospective	63 ± 6	40	8.12 ng/mL (7.56)	Staging	None	68Ga-PSMA-11	40	Detection of disease	PSMA PET/MRI and mpMRI perform equally for the detection of the primary tumor in intermediate-high-risk PCa patients.
Domachevsky et al.	[55]	2020	Israel	Retrospective	69.4 ± 9.3	26	NA	Staging and restaging	NA	68Ga-PSMA-11	26	Technical aspect	Five compartmental model may alter the evaluation of SUV.
Kranzbuhler et al.	[56]	2020	Switzerland	Retrospective	65 (10)	66	0.23 ng/mL (0.03–0.5)	Restaging	RP, ADT and RT	68Ga-PSMA-11	66	Detection of disease	PSMA PET/MRI has a detection rate of 54.5% for a PSA < 0.5 ng/mL. It can change the RT planning in 39.4%.

*RA*, radiopharmaceutical agent; *NA*, not available; *IQR*, interquartile range; *RP*, radical prostatectomy; *RT*, radiotherapy; *ADT*, androgen deprivation therapy; *HIFU*, high intensity focused ultrasound; *CT*, chemotherapy; *BRT*, brachytherapy; *PCa*, prostate cancer; *ADC*, apparent diffusion coefficient; *mpMRI*, multiparametric magnetic resonance imaging; *SUV*, standardized uptake value; *SFCM/SVM*, spatially constrained fuzzy c-means algorithm/support vector machine; *BCR*, biochemical recurrence; *PI-RADS*, prostate imaging reporting and data system; *ePLND*, extensive pelvic lymph node dissection

### Methodological quality

**Fig. 2:**
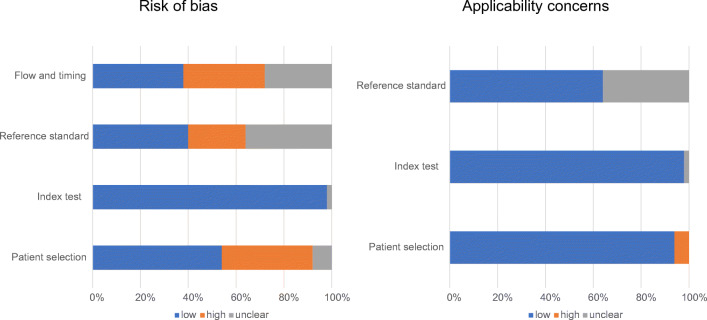
QUADAS-2 findings on the qualitative assessment of the studies selected

All 50 studies were assessed with the QUADAS-2 tool (Fig. 2). The risk of bias for patient selection was high in many papers [10, 15, 19–21, 23, 24, 31, 37, 40–42, 44, 49–51, 53, 55]. The flow and timing were also high in 17 studies [10, 15, 20–24, 26, 27, 31, 32, 35, 44, 45, 51–53]. The applicability of the studies was adequate in most cases, but unclear as regards the reference standard in 18 of them [15, 22–24, 27, 34–37, 41–44, 49, 50, 52, 53, 56].

### PET/MRI for initial staging

In the present review, 15 studies dealt with PET/MRI used only in the staging setting for the purpose of detecting primary disease [11, 12, 14, 18, 29, 31, 33, 35, 38, 40, 44–47, 54, 57].

Integrated PET/MRI proved to be of greater diagnostic value in locating PCa than either multiparametric (mp) MRI [11, 14, 18, 29, 35, 44, 45, 54, 58] or PET imaging alone [14, 18, 44]. 68Ga-PSMA-11 PET/MRI showed high lesion contrast and an excellent consistency in lesion detection [20]. Intense ^18^F-labelled PSMA uptake on PET and mpMRI changes correlated strongly with the dominant lesion in the prostate glands of men undergoing imaging before surgery [31]. These results are consistent with other studies where PET was used to identify PCa lesions. For instance, Park et al. [38] reported that PCa was detected by ^68^Ga-PSMA-11 PET in all of their 33 patients, whereas mpMRI with the PI-RADS (Prostate Imaging Reporting and Data System) pinpointed 4 or 5 lesions in 26 patients, but missed tumors in 3. Similarly, Ferrero et al. [47] found primary tumors PSMA-negative in only 3 of 60 patients, thus reaching a detection rate of 95%.

The assessment of extracapsular extension, tumor grade, and Gleason score plays an important part in treatment decisions, and in distinguishing aggressive from indolent disease. In one study, extracapsular spread of PCa was detected better with ^68^Ga-PSMA-11 PET/MRI than with mpMRI (69 vs. 46%) [54]. In another study, PET and PET/MRI produced a considerably lower proportion of equivocal results (i.e., PI-RADS 3) than mpMRI [35].

PET/MRI may have also an important role in detecting local and distant metastases. From a visual inspection of 60 patients’ imaging results, ^68^Ga-PSMA-11 PET/MRI revealed positive lymph nodes in 8 patients, with only one patient subsequently resulting false-positive. Most nodes were located in the pelvis, but distant nodes were found in the common iliac chain in 2 patients [35]. ^68^Ga-PSMA-11 PET/MRI provides valuable diagnostic information and improves patient selection for extended pelvic lymph node dissection by comparison with the currently-used clinical nomograms [38, 40, 47].

The rate of changes to patient management can express the impact of PET/MRI on the initial staging of PCa patients. Grubmuller et al. [46] reported that including PET/MRI in the initial workup of patients with PCa could alter the therapeutic strategy in at least 30% of cases.

### PET/MRI in cases of biochemical disease recurrence

PET/MRI was used to seek biochemical recurrences of PCa in a total of 598 patients [8, 26, 32, 34, 39, 42, 51, 52, 56]. Taking the studies concerned together, the recurrent disease detection rate achieved with PET/MRI ranged between 54.5 [56] and 97% [8] (Table 2). In many cases, the authors also reported the detection rate by PSA category, which rose with antigen levels from low (< 0.2 ng/mL) to high (> 10 ng/mL). Hope et al. [26] reported a detection rate of 58–64% for PSA levels <0.5 ng/mL using 68Ga-PSMA-11 PET/MRI, while it was 100% for PSA > 2.0 ng/mL [26]. Grubmuller et al. [34] confirmed as much. A number of authors [26, 28, 34, 51] detected a change in patient management prompted by PET/MRI findings, in proportions of cases ranging from 53.2 to 74.6%. Based on the study by Kranzbuhler et al. [56], including PET/MRI in the diagnostic workup could prompt changes to radiotherapy planning for 39.4% of patients.

**Table 2:** Detection rates of PET/MRI in restaging

Authors	Ref	*N* of pts	Detection rate
Afshar-Oromieh et al.	[59]	20	80%
Freitag et al.	[30]	119	78.2%
Lütje et al.	[25]	25	89.6%
Hope et al.	[26]	150	82% PSA level: 58% (< 0.2 ng/mL) 64% (0.2–0.5 ng/mL) 64% (0.5–1 ng/mL) 67% (1–1.5 ng/mL) 100% (1.5–2 ng/mL) 93% (2–5 ng/mL) 93% (> 5 ng/mL) PSAdt: 83% (0–3 months) 90% (3–6 months) 97% (6–12 months) 88% (> 12 months)
Eiber et al.	[21]	75	84.7% (team readers 1) 85.3% (team readers 2)
Lake et al.	[23]	55	89.1% PSA level: 75% (0–1 ng/mL) 80% (1–2 ng/mL) 94.6% (≥2 ng/mL)
Kranzbühler et al.	[32]	56	78.6% PSA level: 44.4% (< 0.2 ng/mL) 72.7% (0.2–< 0.5 ng/mL) 80% (0.5–< 2 ng/mL) 95.2% (≥ 2 ng/mL)
Grubmüller et al.*	[34]	117	85.5% PSA level: 65% (0.2 to < 0.5 ng/mL) 85.7% (0.5–< 1 ng/mL) 85.7% (1–< 2 ng/mL) 100% (≥ 2 ng/mL)
Riola-Parada et al.	[39]	27	55.56% PSA level: 42.86% (< 1 ng/mL) 0% (1–1.9 ng/mL) 75% (2–2.9 ng/mL) 71.43% (3–3.9 ng/mL) 60% (≥ 4 ng/mL)
Ferda et al.	[42]	100	94% PSA level: 33.3% (< 0.2 ng/mL) 88.89% (0.2–2 ng/mL) 97.96% (2.1–5 ng/mL) 100% (5.1–10 ng/mL) 100% (≥10.1 ng/mL)
Achard et al.	[51]	58	58.6% PSA level: 12.5% (< 0.5 ng/mL) 42.9% (0.5–1 ng/mL) 60% (1–2 ng/mL) 85.7% (≥ 2 ng/mL)
Abufaraj et al.	[8]	65	97%
Kranzbuhler et al.	[56]	66	54.5% PSA level: 38.5% (< 0.2 ng/mL) 65% (0.2–0.5 ng/mL)

*PSA*, prostate-specific antigen; *dt*, doubling time

^*^Both PET/MRI and PET/CT

### PET/CT vs. PET/MRI

PET/MRI and PET/CT were compared in seven studies (Table 3; [10, 16, 20, 21, 25, 30, 59] encompassing 278 examinations, 225 of them using ^68^Ga-PSMA-11 (81%) and 53 with ^11^C-choline (19%).

**Table 3:** Detection rates for radiolabeled PSMA and Choline PET/CT vs. PET/MRI in Prostate Cancer

*N*	Authors	Ref	Type of analysis	Detection rate PET/CT	Detection rate PET/MRI
1	Afshar-Oromieh et al.	[59]	Lesion-based	74/75 (99%)	69/75 (92%)
2	Souvatzoglout et al.	[10]	Lesion-based	79/80 (99%) LR: 19 LN: 42 DM: 18	77/80 (96%) LR: 20 LN: 40 DM: 17
3	Freitag et al.	[16]	Patient-based	LR: 9/119 (8%)	18/119 (16%)
4	Domachevsky et al.	[20]	Lesion-based	63/63 (100%)	61/63 (97%)
5	Eiber et al.	[21]	Patient-based Lesion-based (R-1) Lesion-based (R-2)	58/75 (77%) 155/188 (82%) LR: 24 LN: 74 DM: 57 160/188 (85%) LR: 36 LN: 72 DM: 62	63/75 (84%) 148/188 (79%) LR: 36 LN: 60 DM: 52 143/188 (76%) LR: 32 LN: 60 DM: 51
6	Lutje et al.	[25]	Lesion-based	36/46 (78%) LR: 9 LN: 20 DM: 7	43/46 (93%) LR: 14 LN: 23 DM: 6
7	Freitag et al.	[30]	Lesion-based	89/90 (99%)	90/90 (100%)

*LR* local recurrence, *LN* lymph node, *DM* distant metastasis; *R* reader

The overall discrepancy in PET-positive findings between PET/CT and PET/MRI was very low, and agreement between the two methods was high, in the range of 71 to 95% [20, 30, 60]; this also was applied to the semiquantitative analyses [10, 30].

Five studies demonstrated that PET/MRI was superior to PET/CT in detecting PCa lesions, both in staging and restaging [16, 21, 25, 30, 59]. In particular, PET/MRI was more accurate than PET/CT in detecting local recurrences, thereby improving the detection rate for lower PSA levels. All authors [16, 21, 25, 30, 59] found the MRI component crucial in identifying local recurrences otherwise masked by the accumulation of the radiopharmaceuticals in the bladder, especially when ^68^Ga-PSMA-11 was used.

Regarding the assessment of lymph node involvement, PET/MRI achieved a slightly higher detection rate than PET/CT, probably due to a longer tracer accumulation time, as mentioned in the studies by Freitag et al. [16] and Lutje et al. [25].

As for identifying bone metastases, Eiber et al. [21] argue that PET/CT and PET/MRI are comparable for PSA levels < 2 ng/mL, and that PET/CT is more efficient for levels > 2 ng/mL. Freitag et al. [16] and Souvatzoglou et al. [10] claim instead that using multiple MRI sequences improves the detection of bone metastases, especially in cases of early bone marrow involvement.

PET/MRI demands a 79.7% (range, 72.6–86.2%) lower exposure to radiation than PET/CT [21, 59], but the acquisition time is much longer (60 vs. 20 min) [21]. This latter aspect is relative to the inclusion of a mpMRI of the prostate/prostatic fossa that improve significantly the resolution of prostate scan.

### PET/MRI vs. mpMRI

Some papers compared the PCa detection rate or diagnostic performance of PET/MRI and mpMRI in terms of sensitivity and specificity (Table 1s; [13, 14, 18, 29–33, 35, 38, 45, 51, 52, 61]). PET/MRI achieved a higher primary tumor detection rate than mpMRI [14, 18, 45]. Judging from the data reported by de Perrot et al. [13] and Muehlematter et al. [41], PET/MRI was more sensitive than mpMRI in identifying primary tumor in the peripheral zone of prostate gland, and in revealing extracapsular extension and seminal vesicle infiltration. On the other hand, mpMRI provided more information about disease recurrence in the prostatic fossa [30, 51]. As for the detection of lymph node and distant metastases, PET/MRI was more sensitive than mpMRI, in both staging [38] and restaging [32, 51].

### Radiolabeled PSMA vs. radiolabeled choline PET/MRI

The most papers included radiolabeled PSMA as radiopharmaceutical agent. The majority of them were focused on 68Ga-PSMA-11 (*n* = 32 studies), while 2 were based on 18F-PSMA [31, 33]. Radiolabeled choline PET/MRI was employed in the staging for 8/16 (50%) [11–14, 17, 29, 43, 53], while radiolabeled PSMA in 16/34 (47%) papers [16, 18, 27, 31, 35–37, 40, 44–49, 54]. Conversely, 5/16 (31%) [9, 21, 39, 42, 51] and 11/34 (32%) articles [8, 23, 25, 26, 28, 30, 32, 34, 52, 56] were focused on the resting phase for radiolabeled choline and PSMA, respectively.

For the identification of primary lesion, PSMA PET/MRI enriched a specificity of 88%, according to Hicks et al. [45], while choline PET/MRI registered a specificity equal to 76% [13]. Therefore, PSMA is more accurate in detecting primary PCa lesions, by reducing the rate of falsely positive findings. In restaging, PSMA PET/MRI showed a detection rate of 64% for PSA values < 0.5 ng/mL in 150 patients [26], therefore significantly higher than choline PET/MRI (detection rate of 12.5% in 58 patients for the same values of PSA) [51] (see Table 2).

However, no comparative data are now available about radiolabeled PSMA and choline PET/MRI in the same population, in each phase of disease (i.e., staging or restaging).

### Other aspects explored

Six articles considered the image acquisition protocol [19, 23, 24, 37, 48, 53], four discussed technical aspects [15, 22, 41, 55], and eight focused on the interpretation of images obtained with PET/MRI [9, 12, 17, 27, 36, 43, 49, 50].

The best time per bed acquisition using PET/MRI for PCa is longer than 3 min [19, 23] because this can reduce the halo artifact in the bladder and kidney for ^68^Ga-PSMA-11 [24]. According to Heußer et al. [22], the halo artifact can also be reduced by lowering the maximum scatter fraction rate.

The choice of particular MRI sequences has an important influence on the detection of local and distant metastases, as suggested by Metser et al. [53].

The correlation between the apparent diffusion coefficient (ADC) and the standardized uptake value (SUV) is controversial. Wetter et al. [9] found an inverse correlation between ADC and SUV in bone metastases. Uslu-Besli et al. [49] and Tseng et al. [43] likewise reported an inverse correlation between the maximum SUV and the metabolic tumor volume, between uptake volume product and the ADC in primary tumor, respectively. Wetter et al. [12], on the other hand, found no correlation between ADC and SUV in primary cancer.

### Quantitative results

A meta-analysis was performed on 23 studies (Fig. 1), 11 concerning the staging phase [16, 18, 27, 35, 38, 40, 44–47, 52], and 12 the restaging phase [8, 21, 23, 25, 26, 30, 32, 39, 42, 51, 56, 59]. Pooled sensitivities and specificities were obtained for the former (staging), and a pooled detection rate was computed for the latter (restaging).

Table 4 shows the pooled sensitivities and specificities for primary PCa and lymph node disease, showing a higher pooled sensitivity for primary lesions in the patient-based analysis (94.9% [95% CI 87.5–98.6]) than in the lesion-based analysis (61.5% [95% CI 40.6–79.8]). Vice versa, the pooled specificity was higher in the lesion-based analysis than in the patient-based analysis (90.9% [95% CI 80–97] vs. 62.5% [95% CI 43.7–78.9], respectively). For lymph node disease, the pooled sensitivity and specificity were similar in the two types of analysis. The heterogeneity between the studies ranged between 0 and 98.3%.

**Table 4:** Pooled sensitivity and specificity for staging

Site of disease (type of analysis)	Pooled sensitivity (95% CI)	Heterogeneity (*P* value)	*I*-square (%)	Pooled specificity (95% CI)	Heterogeneity (*P* value)	*I*-square (%)
Primary tumor (per-lesion)	61.5% (40.6–79.8)	0.39 (0.531)	0	90.9% (80–97)	8.05 (0.005)	87.6
Primary tumor (per-patient)	94.9% (87.5–98.6)	3.14 (0.076)	68.2	62.5% (43.7–78.9)	0.32 (0.571)	0
Primary tumor (sextant-based)	79.3% (76–82.3)	68.28 (< 0.005)	98.3	83.4% (80.2–86.3)	27.16 (< 0.005)	96.3
Lymph node metastases (per-lesion)	64.3% (44.1–81.4)	2.85 (0.091)	64.9	97.4% (91–99.7)	3.91 (0.048)	74.4
Lymph node metastases (per-patient)	66.7% (49.8–80.9)	0.58 (0.748)	0	93.4% (87.5–97.1)	37.12 (< 0.005)	94.6

*CI* confidence interval

At restaging, the pooled detection rate was 80.9% (95% CI 73.0–86.9%) (Table 5). The pooled detection rate was higher for studies using PET/MRI with radiolabeled PSMA than for those with radiolabeled choline (81.8 vs. 77.3%). The heterogeneity between the studies was high (> 80%). There was also evidence of publication bias, as illustrated by the funnel plot (Supplemental Figure 1).

**Table 5:** Pooled detection rate in restaging

	Pooled detection rate (95% CI)	Heterogeneity (*P* value)	*I*-square
All reports	80.9% (73.0–86.9)	59.531 (< 0.005)	81.522
PSMA PET/MRI	81.8% (72.4–88.4)	35.014 (< 0.005)	80.008
Choline PET/MRI	77.3% (53.7–90.9)	24.508 (< 0.005)	87.759
PET/CT vs. PET/MRI	95.4% (87.0–98.5) 93.9% (85.4–97.6)	28.222 (< 0.005) 28.812 (< 0.005)	82.283 82.646

*CI* confidence interval

In the studies that compared PET/CT with PET/MRI in the same population, the pooled detection rates were 95.4% (95% CI 87.0–98.5) and 93.9% (95% CI 85.4–97.6), respectively; and, here again, the heterogeneity among the studies was > 80%.

## Discussion and conclusions

The data emerging from the available literature suggest some considerations.

1. The ability of PET/MRI with radiolabeled PSMA to detect dominant lesions (pooled sensitivity for sextant-based analysis, 80%) may suggest a further search on prostate fusion biopsy of the suspected area. A recent paper by Westphalen et al. [62] reported a low positive predictive value (PPV) of PI-RADS for identifying primary PCa. After reviewing mpMRI images from 3449 patients for a total of 5082 lesions, the authors found a PPV of 5% for PI-RADS 2, 15% for PI-RADS 3, 39% for PI-RADS 4, and 72% for PI-RADS 5. Park et al. [38] found that PET/MRI with 68Ga-PSMA-11 had a higher PPV than mpMRI for bilateral tumors (70 vs. 18%, respectively). Two articles discussed about the role of PET/MRI for the diagnosis of PCa. Taneja et al. [37] and Jena et al. [44] showed that dual-phase simultaneous 68Ga-PSMA-11 PET/MRI is able to characterize prostate lesions, in 117 patients. In particular, Taneja et al. reported that malignant lesions have higher PSMA uptake than the benign ones, mainly in the delayed images (acquired after about 50 min form tracer injection) due to a possible role of receptor density and longer retention of PSMA in PCa over time. Moreover, Jena et al. [44] concluded that combining PET data, MRI data, PSA levels, and digital rectal examination resulted in a better characterization of prostatic lesions, with an AUC of 0.94 ± 0.29. However, in the setting of primary PCa, MRI-TRUS fusion biopsy using mpMRI will remain the standard for prostate cancer probably for longer time due to a very high-quality study [63]. Similar studies for PSMA PET/MRI-guided biopsy are needed to compete with mpMRI in order to elucidate the advantages in terms of diagnostic efficiency and costs.
  Although the detection of more lesions by use of PET/MRI in primary setting may not necessarily lead to better outcome in general, the identification of oligometastasic disease would be useful for guiding to an appropriate treatment management (extension of the radiation field, extension of lymph node adenectomy, etc.) therefore allowing a long-term prognosis of the patients.
2. Targeted therapies could be directed by PET/MRI with radiolabeled PSMA because of its ability both to detect the most aggressive lesion and to assess the extracapsular extension of disease. This latter information would be useful not only to guide to more precise surgical approach, but it can be useful for focal or less-invasive treatments.
3. PET/MRI with radiolabeled PSMA could be used for early disease recurrences (PSA levels < 0.5 ng/mL) because it can raise the detection rate to 65% and could also be helpful in guiding MDT. It seems that mpMRI can suffice for identifying PCa recurrences in the prostatic fossa. However, the added value of PET/MRI is its ability to detect also the lymph node involvement thus guiding to a specific salvage therapy, especially in case of radiotherapy. Furthermore, in case of a positivity only in the lymph node, a salvage lymph node dissection can be planned, by evaluating also the possible nerve or other neighboring structure involvement.
4. PSMA PET/MRI is more detectable than choline PET/MRI in staging and in restaging, although head to head comparative data are missing.
5. PSMA PET/MRI can prompt changes to the management of PCa patients in up to 75% of cases at restaging. It means that in population of 100 patients with a PCa, the inclusion of PET/MRI in the diagnostic algorithm has a deep effect on the management and therefore on the short- and long-term prognosis. However, more data are necessary for this latter indication, being the literature scarce.

This hybrid imaging modality has some limitations, however, such as the need for scatter correction and long acquisition times. The accurate description and interpretation of the results are also key challenges for radiologists/specialists in nuclear medicine and urologists alike.

In short, PET/MRI seems to have potential applications in the following: (1) the diagnosis of primary tumor; (2) facilitating biopsy targeting; (3) predicting or monitoring tumor aggressiveness (especially during active surveillance); (4) the early detection of recurrent PCa; and (5) guiding targeted therapies.

## Electronic supplementary material

ESM 1 (DOCX 18 kb).

ESM 2 (PPTX 1166 kb).
