# Supplementary material for: PET/MRI in prostate cancer: a systematic review and meta-analysis
Source: Eur J Nucl Med Mol Imaging. 2020 Sep 8;48(3):859–73. doi: 10.1007/s00259-020-05025-0 (PMC8036222; doi:10.1007/s00259-020-05025-0)
Supplement: Supplementary file 1 — (DOCX 18 kb). [file 259_2020_5025_MOESM1_ESM.docx]

**Table 1S.** mpMRI and PET/MRI comparison in selected studies

| **Author** | **Ref** | **N of pts** | **Setting** | **mpMRI** | | **PET/MRI** | |
| --- | --- | --- | --- | --- | --- | --- | --- |
|  |  |  |  | **Detection rate**  **(or DP)** | **Detection rate per site** | **Detection rate (or DP)** | **Detection rate per site** |
| de Perrot et al | 57 | 26 | Staging | PZ+TZ: Sens 66%  TZ: Sens 83.3%  PZ: Sens 75.6% | T: na  N: na  M: na | PZ+TZ: Sens 79.2%  TZ: Sens 58.8%  PZ: Sens 87.6% | T: na  N: na  M: na |
| Kim et al | 39 | 30 | Staging | 25/30 (83.3%) | T: 25 (83.3%)  N: na  M: na | 28/30 (93.3%) | T: 28 (93.3%)  N: na  M: na |
| Eiber et al | 40 | 53 | Staging | 35/53 (66%) | T:35 (66%)  N: na  M: na | 52/53 (98%) | T:52 (98%)  N: na  M: na |
| Lee et al | 41 | 31 | Staging | Sens 58%  Spec 87.3%  Acc 66.7% | T: na  N: na  M: na | Sens 68.7%  Spec 72.7%  Acc 69.9% | T:  N:na  M:na |
| Freitag et al | 55 | 93 | Restaging | 18/119 (15.1%) | T: 15.1% (r)  N: na  M: na | 9/119 (7.6%) | T: 7.6% (r)  N: na  M: na |
| Bauman et al | 15 | 6 | Staging | 6/6 (100%) | T: 6 (100%)  N: na  M: na | 6/6 (100%) | T: 6 (100%)  N:  M: |
| Kranzbühler et al | 29 | 56 | Restaging | 13/54 (24%) | T: 3  N: 5  M: 1 | 43/56 (76%) | T: 10  N: 33  M: 11 |
| Freitag et al | 42 | 8 | Staging Restaging |  | T: 7/7 (100%)  N (r): 0/1 (0%) |  | T: 7/7 (100%)  N (r):1/1 (100%) |
| Al-Bayati et al | 30 | 22 | Staging | 54% per lesions | T: 22/41 lesions  N: na  M: na | 56% per lesion | T: 23/41 lesions  N: na  M: na |
| Park et al | 43 | 33 | Staging |  | T: 26/33 (78.8%)  N: 0/33 (0%)  M: na |  | T: 33/33 (100%)  N: 7/33 (21%)  M: |
| Muehlematter et al | 18 | 20 | Staging Restaging | ECE sens: 0.67  SVI sens: 0.66 | T: na  N: na  M: na | ECE sens: 0.75  SVI sens: 0.74 | T: na  N: na  M: na |
| Hicks et al | 31 | 32 | Staging | 23/29 (79%) | T: 79%  N: na  M: na | 31/32 (97%) | T: 97%  N: na  M: na |
| Achard et al | 23 | 53 | Restaging | 32/58 studies (55%) | T: 17 (29%)  N: 9 (15%)  M: 6 (10%) | 42/58 studies (72%) | T: 14 (21%)  N: 16 (27%)  M: 12 (21%) |
| Burger et al | 32 | 10 | Restaging | all negative | T: 0  N: na  M: na | 6/10 | T: 60%  N: na  M: na |

DP: diagnostic performance; Mp:multiparametric; T:tumor; N;nodal; M:metastasis; na=not available; TZ: transitional zone; PZ: peripheral zone; r=recurrent; ECE: extracapsular extension: SVI: seminal vesicle infiltration
