# Supplementary material for: PET/MRI in prostate cancer: a systematic review and meta-analysis
Source: Eur J Nucl Med Mol Imaging. 2020 Sep 8;48(3):859–73. doi: 10.1007/s00259-020-05025-0 (PMC8036222; doi:10.1007/s00259-020-05025-0)
Supplement: Supplementary file 2 — (PPTX 1166 kb). [file 259_2020_5025_MOESM2_ESM.pptx]

## Slide 1
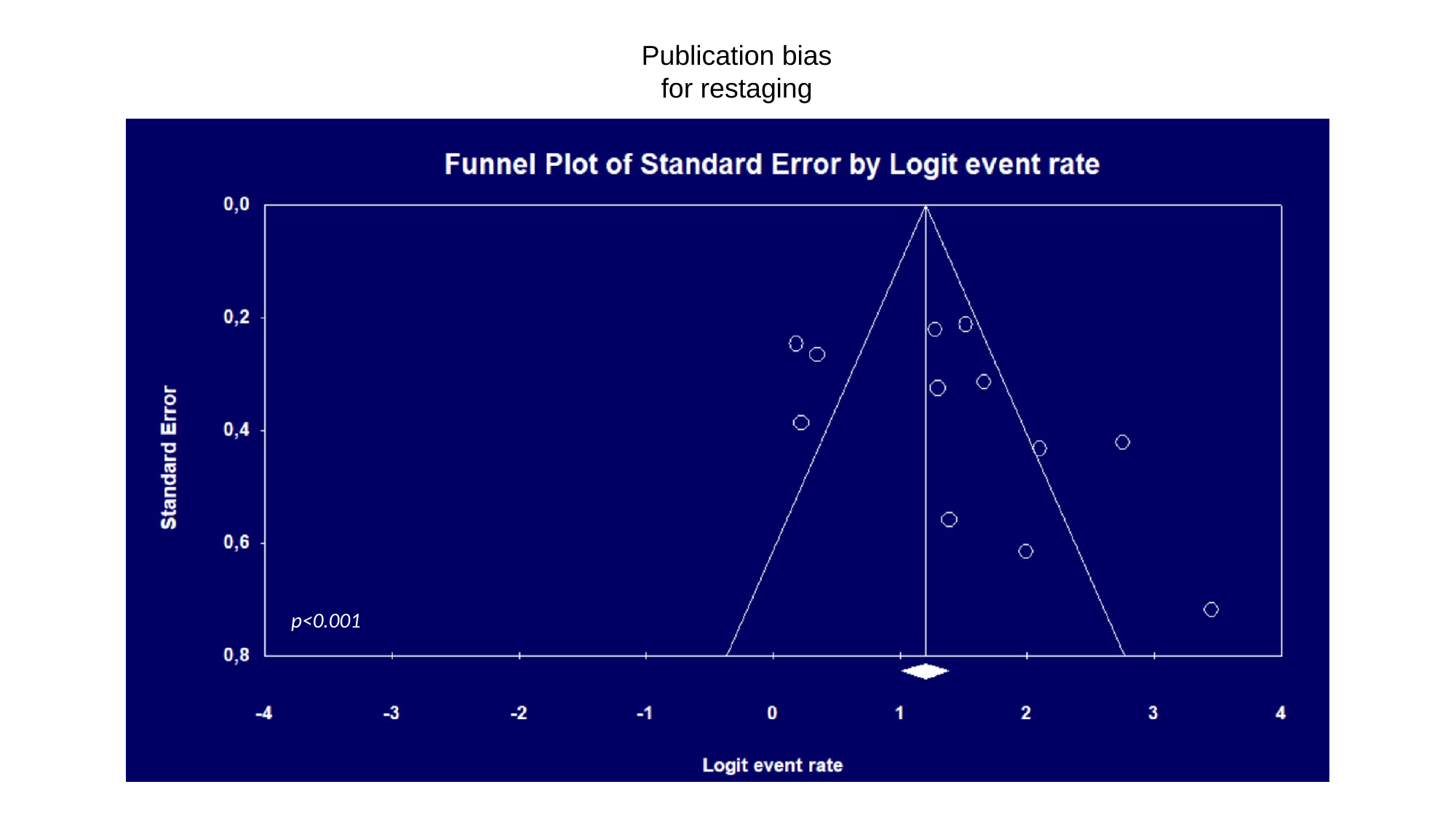

Publication bias for restaging
p<0.001

## Slide 2
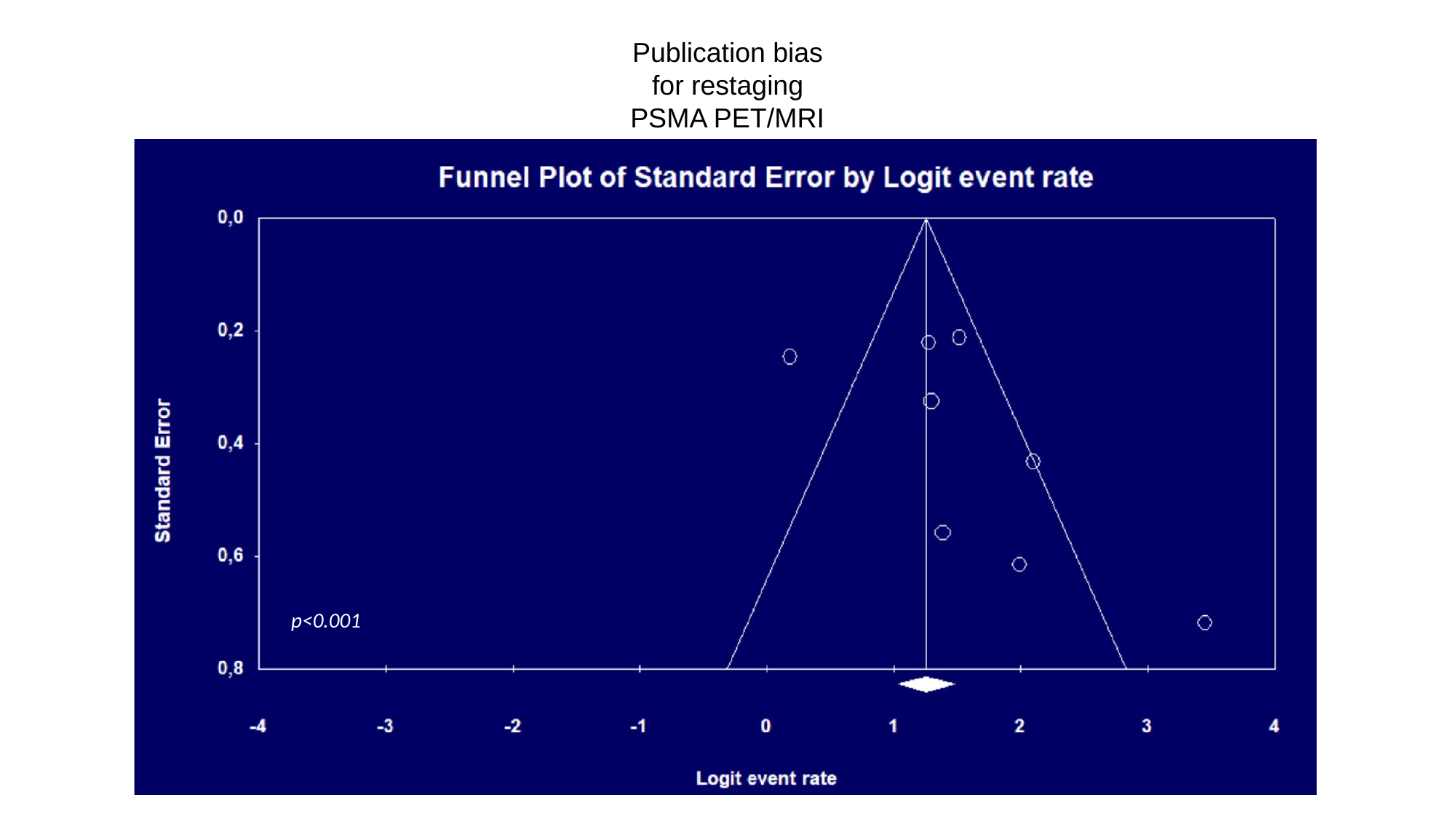

Publication bias for restaging
PSMA PET/MRI
p<0.001

## Slide 3
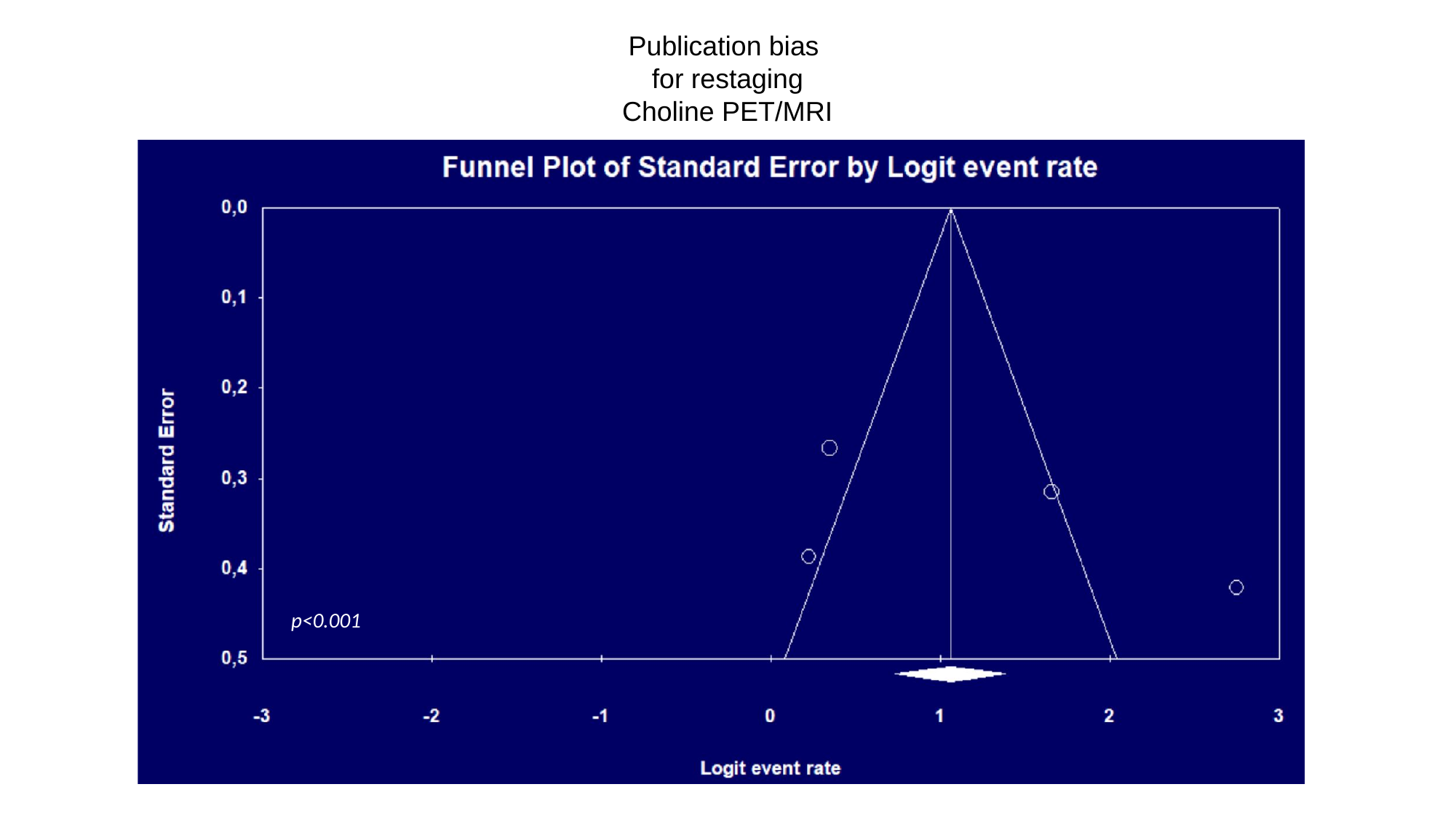

Publication bias
for restaging
Choline PET/MRI
p<0.001
